# Using a data-driven approach to define post-COVID conditions in US electronic health record data

**DOI:** 10.1371/journal.pone.0300570

**Published:** 2024-04-05

**Authors:** Kathleen M. Andersen, Farid L. Khan, Peter W. Park, Timothy L. Wiemken, Birol Emir, Deepa Malhotra, Tuka Alhanai, Mohammad M. Ghassemi, Leah J. McGrath

**Affiliations:** 1 Vaccines Real World Evidence, Pfizer Inc, New York, New York, United States of America; 2 Global Medical Affairs, Pfizer Inc, New York, New York, United States of America; 3 Global Biometrics and Data Management, Pfizer Inc, New York, New York, United States of America; 4 Ghamut Corporation, East Lansing, Michigan, United States of America; King’s College London, UNITED KINGDOM

## Abstract

**Objective:**

To create a data-driven definition of post-COVID conditions (PCC) by directly measure changes in symptomatology before and after a first COVID episode.

**Materials and methods:**

Retrospective cohort study using Optum® de-identified Electronic Health Record (EHR) dataset from the United States of persons of any age April 2020-September 2021. For each person with COVID (ICD-10-CM U07.1 “COVID-19” or positive test result), we selected up to 3 comparators. The final COVID symptom score was computed as the sum of new diagnoses weighted by each diagnosis’ ratio of incidence in COVID group relative to comparator group. For the subset of COVID cases diagnosed in September 2021, we compared the incidence of PCC using our data-driven definition with ICD-10-CM code U09.9 “Post-COVID Conditions”, first available in the US October 2021.

**Results:**

The final cohort contained 588,611 people with COVID, with mean age of 48 years and 38% male. Our definition identified 20% of persons developed PCC in follow-up. PCC incidence increased with age: (7.8% of persons aged 0–17, 17.3% aged 18–64, and 33.3% aged 65+) and did not change over time (20.0% among persons diagnosed with COVID in 2020 versus 20.3% in 2021). For cases diagnosed in September 2021, our definition identified 19.0% with PCC in follow-up as compared to 2.9% with U09.9 code in follow-up.

**Conclusion:**

Symptom and U09.9 code-based definitions alone captured different populations. Maximal capture may consider a combined approach, particularly before the availability and routine utilization of specific ICD-10 codes and with the lack consensus-based definitions on the syndrome.

## Introduction

The definition of post-COVID conditions (PCC), or “long COVID”, is constantly evolving, but generally describes the wide range of health consequences present for some duration after SARS-CoV-2 infection. Within PCC, there is not yet consensus on which symptoms, severity, and duration define the syndrome. Last updated September 1, 2022, the Centers for Disease Control and Prevention (CDC) in the United States defines PCC as ongoing health problems four weeks or more after initial COVID diagnosis [1]. The CDC lists the most commonly reported conditions as tiredness or fatigue, post-exertional malaise, fever, as well as respiratory and cardiac, neurologic and digestive symptoms. The World Health Organization (WHO) commissioned a Delphi consensus process to develop their clinical case definition, last updated October 6, 2021, which includes 12 domains and begins at 3 months after diagnosis [2]. Different still, the United Kingdom’s National Institute for Health and Care Excellence’s (NICE) rapid guideline, last updated November 11, 2021, developed an alternative case definition, which is similar to the WHO definition but has differences in the time window (4 weeks versus 3 months). Additionally, the NICE guidance evaluated the quality of the evidence base, with most of the 33 domains having low or very low certainty of evidence [3]. Finally, some definitions make a distinction for post-acute sequelae of SARS-CoV-2 infection (PASC) as new diagnoses in persons with resolved infections, including the onset of autoimmune conditions, or end organ damage such as subsequent cardiovascular events, each of which have been previously described [4, 5].

While an ICD-10-CM code for PCC came into use on October 1, 2021, it is not known whether uptake represents the true incidence of PCC as compared to patient-reported measures of PCC such as direct patient surveys. In the first four months of its availability, the code was shown to be used across a range of ages in clinical practice in the United States and concomitant with a broad set of clinical conditions [6]. Further, any work before October 2021 will need to consider alternative approaches for measuring PCC.

### Objective

Given the lack of a standardized definition, our objective was to utilize individual-level clinical and contextual information to define the changes in diagnoses and symptoms as a potential data-driven definition for PCC.

## Materials and methods

### Study setting and population

We performed a retrospective cohort study using the Optum® de-identified Electronic Health Record (EHR) dataset from over 104 million unique lives treated in the United States. Optum^®^ EHR data are sourced from over 760 hospitals and 7,000 clinics, with the majority being integrated delivery networks. Records include information on patient demographics, as well as clinical diagnoses, vital signs and body measurements, laboratory results, procedures performed, and medications prescribed during the encounter.

### Inclusion and exclusion criteria

We required persons to have at least one health system encounter recorded between April 1, 2019 –March 31, 2020 to establish pre-pandemic baseline health status, and to have non-missing values for age. Encounters that did not have any diagnosis codes, such as those for laboratory draws, were not counted as health system encounters.

The COVID population was defined as persons with a health system encounter with ICD-10-CM code of U07.1 “COVID-19” or a positive SARS-CoV-2 polymerase chain reaction (PCR) test result from April 1, 2020 through September 30, 2021. The earliest code or positive lab result was used as the COVID index date. We limited the analysis to the first COVID encounter. We compared the COVID attack rate, or the proportion of persons with an encounter each month who were diagnosed with COVID, to CDC data to examine whether this cohort represented national infection trends [7].

The comparator population was used to provide a baseline for symptom or disease incidence scoring, as well as severity of the clinical incident. This group was designed to be similar to the COVID population, in order to reduce bias. For every COVID person selected, we performed 3:1 nearest neighbor matching without replacement [8]. We used a propensity score to calculate the probability of a given person having COVID, with sex, race, ethnicity, insurance payor, overweight or obesity, history of smoking and Charlson-Deyo Comorbidity Index as the variables and used a caliper of 0.1 standard deviations for matches **(S1 Table)** [9]. We required comparators to have a health system encounter for any reason other than COVID in the same month as their match, to reduce detection bias, and used the closest encounter date to the COVID date as the comparator’s index date. Comparators also had to be no more than one year in age different than the COVID patient. Finally, we required comparators to not have had COVID at any time prior to their index date, however, comparators could later be diagnosed with COVID [10]. Matched pairs were not retained in the analysis. Due to the potential protopathic bias where persons might exhibit signs or symptoms of COVID before formal diagnosis, records for comparators 7 days before COVID diagnosis were not attributed to COVID or comparator estimates.

### Outcomes & follow-up

To identify new or persistent symptoms or diseases, we used ICD-10-CM codes at the subchapter level (first 3 characters). All ICD-10-CM codes were assessed with the exception of Z codes, which are related to health system contact rather than specific medical diagnoses, or codes indicating personal history of disease **(S2 Table)**.

First we quantified the incidence of each ICD-10-CM subchapter per person as the presence or absence of new symptoms. Second, for each ICD-10-CM subchapter, an incidence ratio was calculated as the proportion of people with the symptom in the COVID group divided by the proportion of people with the symptom in the comparator group. Those conditions which were more common in the COVID group than non-COVID group (i.e. incidence ratio > 1) formed the set of ICD-10-CM subchapter codes used in a code list to define PCC in a data-driven manner.

**Fig 1:**
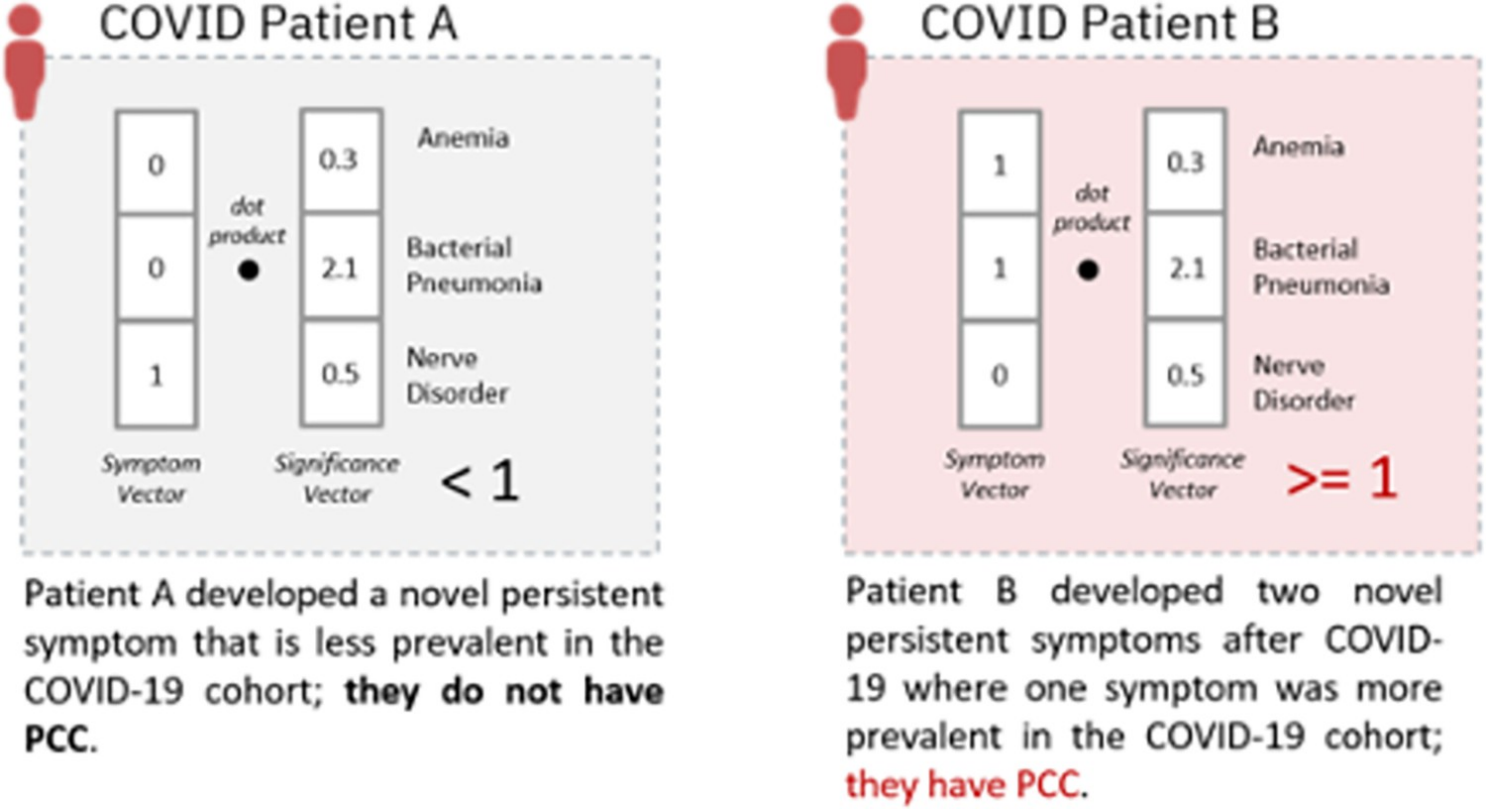
Illustration of how PCC is computed for a given COVID patient using both the patient’s medical profile (symptom vector of novel persistent conditions) and the incidence of the conditions in COVID patients relative to non-COVID patients (significance vector). The patient’s symptom vector and significance vector are combined (via a dot product) to compute whether a COVID patient had PCC.

Finally, in the COVID population only, for each individual the presence of PCC was defined as the dot product of their symptom vector (with 1 for presence or 0 absence of each ICD-10-CM code in the data-driven definition in their medical record) and the symptom significance vector (with > = 1 for ICD-10-CM subchapters that were more common in the COVID group and < 1 for ICD-10-CM subchapters which were less common in the COVID group). This dot product results in a scalar value which is then dichotomized into a binary value; values ≥ 1 indicate the presence of PCC, while values less < 1 indicate the absence of PCC (**Fig 1**). We assigned zeros to codes that were present prior to index, indicating a pre-existing condition rather than a persistent new symptom. We also excluded a code that only appeared during the acute infection phase (days 1–30 after index) that did not reoccur 31 or more days after index, as this does not meet the definition of PCC in the United States as of Fall 2022. Codes that appeared in days 1–30 that were documented again on day 31 or greater (in the patient’s most recent record) were eligible for incidence ratio calculation.

In summary, PCC was defined as the new onset of at least 1 ICD-10-CM subchapter among the set of codes which were more commonly occurring in the COVID population than at background rates, which was not present in the year prior to COVID diagnosis and was present 31 days or later after first COVID diagnosis. Patients were followed from their index date up to 365 days. Follow-up data was available through March 31, 2022 and did not require any minimum follow-up time.

### Statistical analyses

Baseline characteristics were compared, and standardized mean differences (SMD) were used to assess the COVID and non-COVID groups on the measured factors, with a SMD > 10% indicating a significant difference between the groups [11]. We used bootstrapping with Monte Carlo simulations to sample with replacement in order to estimate the uncertainty of symptom incidence statistics and the PCC label.

We stratified results where differences in groups may reflect different probabilities of PCC, including by age and calendar time. Specifically, we stratified by the following age groups: 0–17, 18–64 and 65+ at index. We also stratified by persons diagnosed with COVID in 2020 versus 2021, to consider differences in disease epidemiology, changing availability in vaccines, and emergence of variants.

Finally, for the subset of persons diagnosed with COVID in September 2021, we compared the performance of our PCC definition to the ICD-10-CM code U09.9. We limited to persons diagnosed in September 2021 as these persons were the first cohort to be at risk of PCC as soon as the code became active. We calculated the accuracy to classify the alternate definition for each of the U09.9 and the data-driven definitions. Of note, we do not use the terms sensitivity or specificity to describe the accuracy of either definition relative to each other given the lack of a gold standard definition. We computed the average total variation distance (TVD) between the multivariate characteristics of each of PCC+, PCC-, U09.9+ and U09.9- population samples using bootstrapping. TVDs were used to quantify the degree to which populations resembled each other, with 1 representing completely distinct groups and 0 representing perfect overlap [12, 13]. We used k-nearest neighbors to assess the consistency of the labels assigned by PCC and U09.9. If the definition of PCC, or U09.9, was consistent, then there would be a high proportion of persons with their nearest neighbor having the same label. We present the incidence of ICD-10 subchapters among persons with PCC and U09.9 as demonstrations of the symptomatology identified using either definition.

First, we estimated incidence ratios using more detailed ICD-10 precision, by using the codes at the level of precision in the record such as A41.89 “Other specified sepsis” rather than the subchapter A41 “Other sepsis”. Second, we no longer excluded codes that indicated “history of” and ICD-10 Z codes.

The cohort for this study was constructed using SAS, version 9.4 (SAS Institute, Cary, North Carolina), and analyses were conducted using Dataiku and Python 3.6.8. This study was deemed exempt from Institutional Review Board (IRB) review pursuant to the terms of the U.S. Department of Health and Human Service’s Policy for Protection of Human Research Subjects at 45 Code of Federal Regulations 46.104(d); category 4 exemption (Sterling IRB ID 10412, Atlanta, Georgia).

## Results

**Fig 2:**
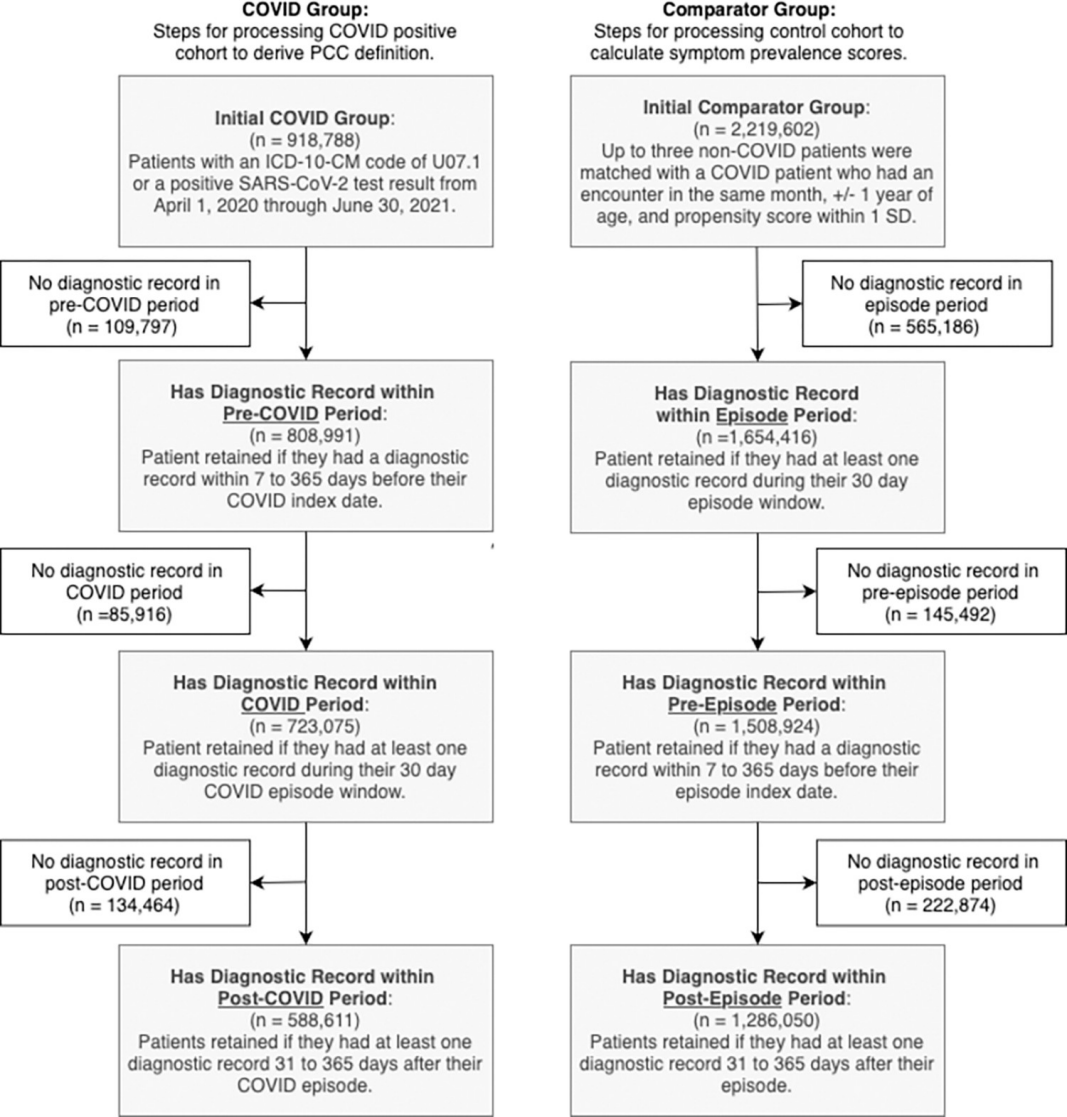
Cohort selection process.

An initial set of 918,788 COVID patients met the inclusion criteria, and a corresponding set of 2,219,602 patients met the inclusion criteria for propensity-matched controls **(Fig 2).** The final cohort contained 588,611 people with COVID and 1,286,050 comparators, creating 1:2.2 sampling. There were 76,698 people in the comparator population who later were diagnosed with COVID; only their pre-COVID records are used for comparator-related calculations.

The groups were well balanced (SMD < 10%) on age, sex, obesity and smoking history, each of which are key risk factors in COVID incidence and severity **(Table 1).**

**Table 1:** Cohort characteristics.

	COVID-19 Positive Cohort	Comparator Cohort	Difference
	(n = 588,611)	(n = 1,286,050)	
Age–years mean (std)	48.1 (20.20)	48.3 (21.2)	0.2
Male	38.4%	40.2%	1.8
Overweight or obesity	68.0%	66.4%	< 0.1
Smoking	41.9%	43.2%	< 0.1
CCI—mean (std)	0.9 (1.6)	0.9 (1.6)	< 0.1
Race: white	76.1%	75.5%	0.6
Race: Black	13.0%	13.5%	0.5
Race: Other/Unknown	9.1%	9.2%	< 0.1
Race: Asian	1.9%	1.9%	< 0.1
Ethnicity: Not Hispanic	82.5%	82.2%	0.3
Insurance: Commercial	71.8%	71.0%	0.8
Insurance: Medicare	11.2%	12.3%	1.1
Insurance: Medicaid	7.6%	7.8%	0.2
Region: Midwest	52.6%	50.3%	2.3
Region: South	18.9%	16.8%	2.1
Region: Northeast	18.9%	20.4%	1.5
Region: West	5.8%	8.7%	2.9

CCI: Charlson Comorbidity Index; std: standard deviation. Continuous variables are shown as mean (standard deviation), and categorical variables as number (%). The absolute value of the standardized difference for percentages and standardized mean difference (SMD) for continuous variables are shown.

The COVID attack rate generally reflected CDC’s publicly available data **(S1 Fig).** There was some separation of the curves in the winter of 2020–2021, with an overall cross-correlation of 0.86 (where 1.00 is perfect).

### Relative incidence of ICD-10 subchapters

Incidence ratios in **Table 2** and **S3** Table quantify the ICD-10-CM subchapters which were more commonly found in the COVID group over follow-up than in the comparator group.

**Table 2:** Ten highest incidence ratios, comparing persons with COVID to age, month and propensity-score matched persons.

ICD-10-CM Subchapter code	Incidence in COVID positive group (%)	Incidence in comparator group (%)	Incidence Ratio	Description of ICD-10-CM Subchapter Code
J12	1.269	0.001	1359.55	Viral pneumonia, not elsewhere classified
J80	0.172	0.001	275.84	Acute respiratory distress syndrome
U09	0.016	0.000	70.64	Post COVID-19 condition
B94	0.068	0.002	41.51	Sequelae of other and unspecified infectious and parasitic diseases
T68	0.003	0.000	32.77	Hypothermia
Y95	0.017	0.001	24.28	Nosocomial condition
J15	0.145	0.006	23.92	Bacterial pneumonia, not elsewhere classified
I76	0.002	0.000	21.85	Septic arterial embolism
J13	0.003	0.000	19.66	Pneumonia due to Streptococcus pneumoniae
J10	0.006	0.000	18.03	Influenza due to other identified influenza virus

For more, see S3 and S4 Tables.

Imbalances were most notable for codes relating to diseases of the respiratory and cardiovascular systems. No clear patterns emerged among the codes less common in COVID patients than comparators **(S4 Table).**

### PCC incidence

Overall, 118,018 persons (20%) developed PCC according to our definition (**Table 3).**

**Table 3:** Incidence of post-COVID conditions, using data-driven definition.

	Population size	Persons with PCC	Incidence of PCC
Overall	588,611	118,018	20.1%
By Age at diagnosis			
0–17	46,039	3,567	7.8%
18–64	414,315	71,810	17.3%
65 or older	128,257	42,641	33.2%
By time periods			
COVID diagnoses 4/1/2020-12/31/2020	349,594	69,608	19.9%
COVID diagnoses 1/1/2021-9/30/2021	239,017	48,410	20.3%
By age and time periods			
Age 0–17, diagnoses 4/1/2020-12/31/2020	21,789	1,618	7.4%
Age 0–17, diagnoses 1/1/2021-9/30/2021	24,250	1,949	8.0%
Age 18–64, diagnoses 4/1/2020-12/31/2020	249,697	41,820	16.8%
Age 18–64, diagnoses 1/1/2021-9/30/2021	164,618	29,990	18.2%
Age 65 or older, diagnoses 4/1/2020-12/31/2020	78,108	26,170	33.5%
Age 65 or older, diagnoses 1/1/2021-9/30/2021	50,149	16,471	32.8%

PCC: Post-COVID Conditions

The proportion of people with PCC increased with age (7.8% age 0–17, 17.3% age 18–64, 33.3% age 65+). The incidence of PCC among persons diagnosed with COVID in 2020 versus 2021 was similar overall (19.9% vs 20.3% respectively) as well as for each age strata.

### Comparison of data-driven definition to ICD-10-CM code U09.9

We evaluated the cross-tabulation between the definition of PCC derived in this work and ICD-10-CM in **Table 4 and S5 Table.**

**Table 4:** Comparison of data-driven definition to ICD-10-CM code U09.9.

Persons diagnosed with COVID in September 2021	U09.9 code present in follow-up	U09.9 code absent in follow-up	
Data-driven PCC present in follow-up	394	5,716	19.0% of cases diagnosed in September
Data-driven PCC absent in follow-up	538	25,538	
	2.9% of cases diagnosed in September		

There were 394 persons out of 32,186 (1.2% of cases in September 2021) who developed PCC according to both the data-driven definition as well as the ICD-10 code. Of the 32,186 persons in the cohort in September 2021, the ICD code identified 2.9% of persons as having PCC, while our definition identified 19.0% of persons (6.6 times as many cases) as having PCC in their follow-up period through March 2022. The accuracy of the definitions to detect the presence of PCC using the alternate method was worse than chance, with 42% of U09.9 cases meeting the data-driven PCC definition and 6% of data-driven defined PCC cases having a U09.9 code. Conversely, the accuracy to rule out PCC was high with 82% of people who did not have a U09.9 code also not classified as having PCC with the data-driven definition and 98% of people who did not have a data-driven definition of PCC lacking a U09.9 code.

Both the TVD **(S2 Fig)** and k-nearest neighbor **(S6 Table)** analyses suggest that persons who do not have a U09.9 diagnosis code in follow-up are a heterogenous group. There was less consistency within the U09.9- group (self TVD = 0.50, nearest neighbor consistent 62.7%) than in each of the other groups: PCC+ (self TVD = 0.32, nearest neighbor consistent 99.7%), PCC- (self TVD = 0.27, nearest neighbor consistent 98.6%), U09.9+ (self TVD = 0.28, nearest neighbor consistent 98.9%). While the PCC definition was built on the presence of symptoms in the clinical record, less than half of persons with the ICD-10 code have a descriptive persistent symptom recorded alongside the U09.9 **(S3 Fig)**.

### Sensitivity analyses

Using ICD-10-CM codes at the level of detail in the record, rather than summarized at ICD subchapters, did not make substantial differences in the incidence estimates (20.4%, vs 20.1% in main analysis) **(S7 Table).** Allowing “history of” or ICD-10 Z codes increased the estimated incidence of PCC (24.1%, vs 20.1% in main analysis).

## Discussion

In this study, the incidence of PCC was lower than in other published reports using data from the same time period. A meta-analysis reported a global estimated pooled incidence of 43% (95% confidence interval 39–46%) [14]. The pooled estimates from studies in the US indicated a lower incidence (31%, 95% confidence interval 21%-43%), with ranges from 9%-52% across non-hospitalized and hospitalized groups, as well as a variety of study designs. Prospective studies with active outcome ascertainment, such as patient surveys, may improve case detection and therefore not represent the incidence estimates from retrospective studies of real-world data. It is therefore not surprising that our PCC incidence is lower than these published works [15–17]. Our estimates are also lower than studies which exclusively reported the risk of PCC in persons who were initially hospitalized with COVID [17, 18]. It has been well-documented that initial disease severity increase the risk of subsequent medical events, such as subsequent cardiovascular diagnoses [4].

A strength of this work is the use of within-month comparisons. Most of the literature to date has shown decreases in the incidence of PCC over time, perhaps owing to changes in the control group rather than true disease epidemiologic phenomenon. Our approach is unique and may present a view of PCC that is less susceptible to calendar period influence.

The examination of cases diagnosed in September 2021 afford the unique opportunity to examine the uptake of the U09.9 code in the first month it became available for use. The majority of persons who received this diagnosis code did not have a corresponding symptom in their medical record to describe the details of the post-COVID condition, such as loss of taste or fatigue. Possible explanations include seeking care outside of the EHR system such as for specialist visits, inconsistent application as physicians become aware of the new code, changes to the SARS-CoV-2 virus and severity of COVID-19 within the fall 2021 and winter 2022 period, as well as evolving understanding of the underlying syndrome (PCC).

This work has similar challenges to other work related to COVID using EHR data from the United States [19]. First, COVID vaccination coverage information is poorly captured, given the distribution system was uncoupled from many sites of ongoing medical care such as mass vaccination sites and local pharmacies. Therefore, COVID vaccination coverage in this database appears much lower than official CDC reports for the same time period, and we were unable to evaluate the role vaccination may play in changing the risk of PCC. Second, the EHR data is not a closed network such that persons may have sought care in other health systems, which would not be reflected in these datasets. The Optum^®^ EHR data are predominately integrated data networks, and indeed 80% of persons in the cohort did have a follow-up encounter within 180 days suggesting ongoing care engagement **(S4 Fig)**. It is possible that prior health conditions diagnosed in other care network were not recorded, which would mean some incidence estimates here reflect prevalence instead. Third, some persons in the comparator cohort may have had prior COVID episodes that went undocumented. While our index infection period ended in September 2021, follow-up continued through March 2022, which spans a period including steep increases in omicron infections as well as transition to at-home testing. Persons in the comparator cohort who tested positive at home and did not report to their physician may have post-COVID symptoms attributed to the comparator cohort, which would mean that the incidence ratios presented here are underestimates of the effect in a truly COVID/non-COVID comparison. Lastly, the propensity score used to create groups of comparable persons may not have been properly specified, and therefore there may be residual or unmeasured confounding in comparisons between these two groups.

## Conclusion

This data-driven approach highlights that patients who have recovered from acute COVID-19 are more susceptible to further acute infections at an incidence in excess of comparators, including viral pneumonias (J12) and ARDS (J80). Here, we directly measured changes in symptomatology to define PCC status. Utilizing EHR records provides greater capture of symptoms than claims data alone, while also offering time-saving efficiencies over prospectively captured data. Future studies of PCC may need to consider a hybrid approach of both symptom-based as well as ICD-10 code definitions in order to maximize capture of persons with the condition.

## Supporting information

S1 Table Propensity score description. (DOCX)

S2 Table ICD-10-CM codes excluded from analysis. (DOCX)

S3 Table One hundred highest incidence ratios, comparing persons with COVID to age, month and propensity-score matched persons. (DOCX)

S4 Table One hundred lowest incidence ratios, comparing persons with COVID to age, month and propensity-score matched persons. (DOCX)

S5 Table Comparison of ICD-10-CM code U09.9 to data-driven definition. (DOCX)

S6 Table Comparison of label consistency via K-nearest neighbor analysis. (DOCX)

S7 Table Results of sensitivity analyses. (DOCX)

S1 Fig COVID case attack rate. (DOCX)

S2 Fig Total variation distance measures of post-COVID conditions and U09.9 populations. (DOCX)

S3 Fig Symptomatology comparison between persons with post-COVID conditions and U09.9 codes. (DOCX)

S4 Fig Time to next encounter in Optum^®^ EHR before and after COVID diagnosis. (DOCX)
