# Supplementary material for: Using a data-driven approach to define post-COVID conditions in US electronic health record data
Source: PLoS One. 2024 Apr 5;19(4):e0300570. doi: 10.1371/journal.pone.0300570 (PMC10997091; doi:10.1371/journal.pone.0300570)
Supplement: S1 Table — (DOCX) [file pone.0300570.s001.docx]

# S1 Table: Propensity Score Description

| **Concept** | **Description** |
| --- | --- |
| Sex | Female  Male  Unknown |
| Race | African American  Asian  Caucasian  Other/Unknown |
| Ethnicity | Hispanic  Not Hispanic  Unknown |
| Insurance payor | Commercial  Medicare  Medicaid  Other payor type  Uninsured  Unknown |
| Overweight or obesity | Any measurement in electronic health record indicating body mass index ≥ 25 kg/m^2^ |
| History of smoking | ICD-10-CM F17.X (Nicotine dependence)  ICD-10-CM O99.33X (Tobacco use disorder complicating pregnancy, childbirth, and the puerperium)  obs_type=SMOKE  Smoking cessation consultation |
| Charlson-Deyo Comorbidity Index | Using Deyo’s weights and Quan (2005) algorithm to identify codes in ICD-10-CM data |

We performed 3:1 nearest neighbor matching, with a caliper of 0.1 standard deviations of the propensity score for matches with additional forced matches on calendar month of encounter and age (within 1 year).
