# Supplementary material for: Using a data-driven approach to define post-COVID conditions in US electronic health record data
Source: PLoS One. 2024 Apr 5;19(4):e0300570. doi: 10.1371/journal.pone.0300570 (PMC10997091; doi:10.1371/journal.pone.0300570)
Supplement: S2 Table — (DOCX) [file pone.0300570.s002.docx]

# S2 Table: ICD-10-CM Codes Excluded from Analysis

| **Z codes (list):** z00, z000, z0000, z00000, z0001, z001, z0010, z00110, z00111, z0012, z00121, z00129, z002, z003, z005, z006, z0070, z0071, z008, z01, z0100, z0101, z01020, z01021, z0110, z01110, z01118, z0112, z0120, z0121, z013, z0130, z0131, z014, z01411, z01419, z0142, z018, z01810, z01811, z01812, z01818, z0182, z0183, z0184, z0189, z02, z020, z021, z022, z023, z024, z025, z026, z0271, z0279, z0281, z0282, z0283, z0289, z029, z036, z0371, z0372, z0373, z0374, z0375, z0379, z03810, z03818, z03821, z03822, z03823, z0389, z041, z042, z043, z0441, z0442, z046, z0471, z0472, z048, z0481, z0489, z049, z050, z051, z052, z053, z0541, z0542, z0543, z055, z056, z0571, z0572, z058, z059, z08, z09, z11, z110, z111, z112, z113, z114, z115, z1151, z1152, z1159, z116, z117, z118, z119, z12, z120, z121, z1210, z1211, z1212, z1213, z122, z123, z1231, z1239, z124, z125, z126, z1271, z1272, z1273, z1279, z1281, z1282, z1283, z1289, z129, z13, z130, z131, z1321, z1322, z13220, z13228, z1329, z1330, z1331, z1332, z1339, z134, z1340, z1341, z1342, z1349, z135, z136, z1371, z1379, z13810, z13811, z13818, z13820, z13828, z1383, z1384, z1385, z13850, z13858, z1388, z1389, z139, z1401, z1402, z141, z148, z150, z1501, z1502, z1503, z1504, z1509, z1581, z1589, z1610, z1611, z1612, z1619, z1620, z1621, z1622, z1623, z1624, z1629, z1630, z1632, z1633, z16341, z16342, z1635, z1639, z170, z171, z1809, z1810, z1811, z1812, z182, z1831, z1832, z1833, z1839, z1881, z1883, z1889, z189, z19, z191, z192, z2001, z2009, z201, z202, z203, z204, z205, z206, z207, z20810, z20811, z20818, z2082, z20820, z20821, z20822, z20828, z2089, z209, z21, z220, z221, z222, z2231, z22321, z22322, z22330, z22338, z2239, z224, z2251, z226, z227, z228, z229, z23, z230, z2801, z2802, z2803, z2804, z2809, z281, z2820, z2821, z2829, z283, z28310, z28311, z2839, z2881, z2882, z2883, z2889, z289, z29, z2911, z2912, z2913, z2914, z293, z298, z299, z300, z30011, z30012, z30013, z30014, z30015, z30016, z30017, z30018, z30019, z3002, z3009, z302, z3040, z3041, z3042, z30430, z30431, z30432, z30433, z3044, z3045, z3046, z3049, z308, z309, z31, z310, z3141, z3142, z31430, z31438, z31440, z31441, z31448, z3149, z315, z3161, z3162, z3169, z317, z3181, z3182, z3183, z3184, z3189, z319, z32, z320, z3200, z3201, z3202, z321, z322, z323, z33, z331, z332, z333, z34, z340, z3400, z3401, z3402, z3403, z3480, z3481, z3482, z3483, z349, z3490, z3491, z3492, z3493, z36, z360, z361, z362, z363, z364, z365, z3681, z3682, z3683, z3684, z3685, z3686, z3687, z3688, z3689, z368a, z369, z370, z371, z372, z373, z374, z3750, z3751, z3761, z3763, z377, z379, z380, z3800, z3801, z381, z382, z3830, z3831, z384, z385, z3862, z3864, z3868, z3869, z388, z39, z390, z391, z392, z3a00, z3a01, z3a08, z3a09, z3a10, z3a11, z3a12, z3a13, z3a14, z3a15, z3a16, z3a17, z3a18, z3a19, z3a20, z3a21, z3a22, z3a23, z3a24, z3a25, z3a26, z3a27, z3a28, z3a29, z3a30, z3a31, z3a32, z3a33, z3a34, z3a35, z3a36, z3a37, z3a38, z3a39, z3a40, z3a41, z3a42, z3a49, z4000, z4001, z4002, z4003, z4009, z408, z409, z411, z412, z413, z418, z419, z421, z428, z43, z430, z431, z432, z433, z434, z435, z436, z437, z438, z439, z44011, z44101, z44102, z44109, z44111, z44121, z44122, z4420, z4422, z4430, z4431, z4432, z448, z449, z45, z45010, z45018, z4502, z4509, z451, z452, z4531, z45320, z45321, z45328, z4541, z4542, z4549, z45811, z45812, z45819, z4582, z4589, z459, z460, z461, z462, z463, z464, z4651, z4659, z466, z4681, z4682, z4689, z469, z471, z472, z4731, z4732, z4733, z478, z4781, z4782, z4789, z48, z4800, z4801, z4802, z4803, z481, z482, z4821, z4822, z4823, z4824, z48280, z48288, z48290, z48298, z483, z4881, z48810, z48811, z48812, z48813, z48814, z48815, z48816, z48817, z4889, z4901, z4902, z4931, z4932, z510, z511, z5111, z5112, z515, z516, z5181, z5189, z52000, z52001, z52008, z52010, z52011, z52018, z52090, z52091, z52098, z5210, z5211, z523, z524, z526, z52810, z52811, z52812, z52813, z52819, z5289, z529, z53, z530, z5301, z5309, z531, z5320, z5321, z5329, z5331, z5332, z5333, z5339, z538, z539, z550, z551, z552, z553, z554, z555, z558, z559, z560, z561, z562, z563, z564, z565, z566, z5681, z5682, z5689, z569, z570, z571, z572, z5731, z5739, z574, z575, z576, z577, z578, z579, z586, z59, z590, z5900, z5901, z5902, z591, z592, z593, z594, z5941, z5948, z595, z596, z597, z598, z5981, z59811, z59812, z59819, z5989, z599, z600, z602, z603, z604, z605, z608, z609, z620, z621, z6221, z6222, z6229, z626, z62810, z62811, z62812, z62813, z62819, z62820, z62821, z62822, z62890, z62891, z62898, z629, z630, z631, z6331, z6332, z634, z635, z636, z637, z6371, z6372, z6379, z638, z639, z640, z641, z644, z650, z651, z652, z653, z654, z655, z658, z659, z66, z6710, z6711, z6720, z6721, z6730, z6731, z6740, z6741, z6790, z6791, z68, z681, z6820, z6821, z6822, z6823, z6824, z6825, z6826, z6827, z6828, z6829, z683, z6830, z6831, z6832, z6833, z6834, z6835, z6836, z6837, z6838, z6839, z6841, z6842, z6843, z6844, z6845, z685, z6851, z6852, z6853, z6854, z69010, z69020, z69021, z6911, z6912, z6981, z6982, z700, z701, z702, z703, z708, z709, z71, z710, z711, z712, z713, z7141, z7142, z7151, z7152, z716, z717, z7181, z7182, z7183, z7184, z7185, z7189, z719, z720, z723, z724, z725, z7251, z7252, z7253, z726, z72810, z72811, z72820, z72821, z7289, z729, z730, z731, z732, z733, z734, z735, z736, z73810, z73811, z73812, z73819, z7382, z7389, z739, z7401, z7409, z741, z742, z743, z748, z749, z750, z751, z752, z753, z754, z755, z758, z759, z76, z760, z761, z762, z763, z764, z765, z7681, z7682, z7689, z77010, z77011, z77012, z77018, z77020, z77021, z77028, z77090, z77098, z77110, z77111, z77118, z77120, z77121, z77122, z77123, z77128, z7721, z7722, z7729, z779, z780, z781, z789, z78999, z79, z790, z7901, z7902, z791, z792, z793, z794, z7951, z7952, z7981, z79810, z79811, z79818, z7982, z7983, z7984, z7989, z79890, z79891, z79899, z800, z801, z802, z803, z8041, z8042, z8043, z8049, z805, z8051, z8052, z8059, z806, z807, z808, z809, z810, z811, z812, z813, z814, z818, z820, z821, z822, z823, z824, z8241, z8249, z825, z8261, z8262, z8269, z8271, z8279, z828, z830, z831, z832, z833, z8341, z8342, z83430, z83438, z8349, z8351, z83511, z83518, z8352, z836, z8371, z8379, z84, z840, z841, z842, z843, z8481, z8482, z8489, z8500, z8501, z85020, z85028, z85030, z85038, z8504, z85040, z85048, z8505, z85060, z85068, z8507, z8509, z8511, z85110, z85118, z8512, z8520, z8521, z8522, z85230, z85238, z8529, z853, z8540, z8541, z8542, z8543, z8544, z8545, z8546, z8547, z8548, z8549, z8550, z8551, z85520, z85528, z8553, z8554, z8559, z856, z8571, z8572, z8579, z85810, z85818, z85819, z85820, z85821, z85828, z85830, z85831, z85840, z85841, z85848, z85850, z85858, z8589, z859, z86, z86000, z86001, z86002, z86003, z86004, z86005, z86006, z86007, z86008, z86010, z86011, z86012, z86018, z8603, z861, z8611, z8612, z8613, z8614, z8615, z8616, z8619, z862, z8631, z8632, z8639, z8651, z8659, z8661, z8669, z867, z86711, z86718, z8672, z8673, z8674, z8679, z870, z8701, z8709, z8711, z8719, z872, z87310, z87311, z87312, z8732, z8739, z87410, z87411, z87412, z8742, z87430, z87438, z8744, z87440, z87441, z87442, z87448, z8751, z8759, z87710, z87718, z87720, z87721, z87728, z87730, z87738, z8774, z8775, z8776, z87790, z87798, z8781, z87820, z87821, z87828, z87890, z87891, z87892, z87898, z880, z881, z882, z883, z884, z885, z886, z887, z888, z889, z89011, z89012, z89019, z89021, z89022, z89029, z89111, z89112, z89119, z89121, z89122, z89129, z89201, z89202, z89209, z89211, z89212, z89219, z89221, z89222, z89229, z89231, z89232, z89239, z89411, z89412, z89419, z89421, z89422, z89429, z89431, z89432, z89439, z89441, z89442, z89449, z89511, z89512, z89519, z89521, z89522, z89529, z89611, z89612, z89619, z89621, z89622, z89629, z899, z90, z9001, z9002, z9009, z9010, z9011, z9012, z9013, z902, z903, z90410, z90411, z9049, z905, z906, z90710, z90711, z90712, z90721, z90722, z9079, z9081, z9089, z91, z9101, z91010, z91011, z91012, z91013, z91014, z91018, z9102, z91030, z91038, z91040, z91041, z91048, z9109, z911, z9111, z91120, z91128, z91130, z91138, z9114, z9115, z9119, z91410, z91411, z91412, z91419, z9142, z9149, z915, z9151, z9152, z918, z9181, z9182, z9183, z91841, z91842, z91843, z91849, z9189, z920, z9221, z9222, z9223, z92240, z92241, z9225, z9229, z923, z924, z9281, z9282, z9283, z9284, z92850, z9289, z930, z931, z932, z933, z934, z9350, z9351, z9352, z9359, z936, z938, z939, z94, z940, z941, z942, z943, z944, z945, z946, z947, z948, z9481, z9482, z9483, z9484, z9489, z949, z950, z951, z952, z953, z954, z955, z95810, z95811, z95812, z95818, z95820, z95828, z959, z96, z960, z961, z9620, z9621, z9622, z9629, z963, z9641, z9649, z965, z9660, z96611, z96612, z96619, z96621, z96622, z96629, z96631, z96632, z96639, z96641, z96642, z96643, z96649, z96651, z96652, z96653, z96659, z96661, z96662, z96669, z9669, z96691, z96692, z96693, z96698, z967, z9681, z9682, z9689, z969, z970, z9710, z9711, z9713, z9714, z9716, z972, z973, z974, z975, z978, z98, z980, z981, z982, z983, z9841, z9842, z9849, z9851, z9852, z986, z9861, z9862, z988, z9881, z98810, z98811, z98818, z9882, z9883, z9884, z9885, z9886, z98870, z98871, z9889, z98890, z98891, z990, z9911, z9912, z992, z993, z998, z9981, z9989 |
| --- |
| **“history” codes (list, excluding Z codes):** o0900, o0901, o0902, o0903, o0910, o0911, o0912, o0913, o09211, o09212, o09213, o09219, o09a0, o09a1, o09a2, o09a3 |

Z codes and codes with a reference to a “history” of the condition (using a string match) were excluded.
