# Supplementary material for: Using a data-driven approach to define post-COVID conditions in US electronic health record data
Source: PLoS One. 2024 Apr 5;19(4):e0300570. doi: 10.1371/journal.pone.0300570 (PMC10997091; doi:10.1371/journal.pone.0300570)
Supplement: S3 Table — (DOCX) [file pone.0300570.s003.docx]

# S3 Table: One Hundred Highest Incidence Ratios, Comparing Persons with COVID to Age, Month and Propensity-Score Matched Persons

| **ICD-10-CM Subchapter Code** | **Incidence in COVID positive group (%)** | **Incidence in comparator group (%)** | **Incidence Ratio** | **Description of ICD-10-CM Subchapter Code** |
| --- | --- | --- | --- | --- |
| J12 | 1.269 | 0.001 | 1359.55 | Viral pneumonia, not elsewhere classified |
| J80 | 0.172 | 0.001 | 275.84 | Acute respiratory distress syndrome |
| U09 | 0.016 | 0.000 | 70.64 | Post COVID-19 condition |
| B94 | 0.068 | 0.002 | 41.51 | Sequelae of other and unsp infectious and parasitic diseases |
| T68 | 0.003 | 0.000 | 32.77 | Hypothermia |
| Y95 | 0.017 | 0.001 | 24.28 | Nosocomial condition |
| J15 | 0.145 | 0.006 | 23.92 | Bacterial pneumonia, not elsewhere classified |
| I76 | 0.002 | 0.000 | 21.85 | Septic arterial embolism |
| J13 | 0.003 | 0.000 | 19.66 | Pneumonia due to Streptococcus pneumoniae |
| J10 | 0.006 | 0.000 | 18.03 | Influenza due to other identified influenza virus |
| J16 | 0.001 | 0.000 | 17.48 | Pneumonia due to oth infectious organisms, NEC |
| J95 | 0.051 | 0.004 | 14.20 | Intraop and postproc comp and disorders of resp sys, NEC |
| I40 | 0.005 | 0.000 | 13.98 | Acute myocarditis |
| D65 | 0.006 | 0.000 | 13.11 | Disseminated intravascular coagulation |
| J93 | 0.057 | 0.005 | 12.27 | Pneumothorax and air leak |
| R65 | 0.179 | 0.015 | 12.20 | Symp and signs specifically assoc w sys inflam and infct |
| T41 | 0.004 | 0.000 | 12.02 | Anesthetics and therapeutic gases |
| J96 | 1.038 | 0.089 | 11.67 | Respiratory failure, not elsewhere classified |
| J14 | 0.003 | 0.000 | 11.65 | Pneumonia due to Hemophilus influenzae |
| G92 | 0.051 | 0.005 | 10.96 | Toxic encephalopathy |
| T42 | 0.012 | 0.001 | 10.92 | Antiepileptic, sedative- hypnotic and antiparkinsonism drugs |
| T70 | 0.001 | 0.000 | 10.92 | Effects of air pressure and water pressure |
| L99 | 0.001 | 0.000 | 10.92 | Oth disorders of skin, subcu in diseases classd elswhr |
| T38 | 0.069 | 0.007 | 9.72 | Hormones and their synthetic substitutes and antag, NEC |
| A41 | 0.319 | 0.033 | 9.72 | Other sepsis |
| O98 | 0.087 | 0.009 | 9.66 | Matern infec/parastc dis classd elsw but compl preg/chldbrth |
| G72 | 0.041 | 0.004 | 9.24 | Other and unspecified myopathies |
| J85 | 0.007 | 0.001 | 8.96 | Abscess of lung and mediastinum |
| F05 | 0.034 | 0.004 | 8.92 | Delirium due to known physiological condition |
| Y71 | 0.001 | 0.000 | 8.74 | Cardiovascular devices associated with adverse incidents |
| A18 | 0.001 | 0.000 | 8.74 | Tuberculosis of other organs |
| O87 | 0.001 | 0.000 | 8.74 | Venous complications and hemorrhoids in the puerperium |
| S26 | 0.001 | 0.000 | 8.74 | Injury of heart |
| R57 | 0.053 | 0.006 | 8.39 | Shock, not elsewhere classified |
| R34 | 0.009 | 0.001 | 7.57 | Anuria and oliguria |
| J18 | 0.368 | 0.050 | 7.41 | Pneumonia, unspecified organism |
| J22 | 0.009 | 0.001 | 7.37 | Unspecified acute lower respiratory infection |
| A08 | 0.012 | 0.002 | 7.07 | Viral and other specified intestinal infections |
| X19 | 0.001 | 0.000 | 6.55 | Contact with other heat and hot substances |
| T76 | 0.001 | 0.000 | 6.55 | Adult and child abuse, neglect and oth maltreat, suspected |
| A80 | 0.001 | 0.000 | 6.55 | Acute poliomyelitis |
| N14 | 0.004 | 0.001 | 6.55 | Drug- & heavy-metal-induced tubulo-interstitial & tublr cond |
| C86 | 0.001 | 0.000 | 6.55 | Other specified types of T/NK-cell lymphoma |
| V95 | 0.001 | 0.000 | 6.55 | Accident to powered aircraft causing injury to occupant |
| B78 | 0.001 | 0.000 | 6.55 | Strongyloidiasis |
| I46 | 0.035 | 0.006 | 6.25 | Cardiac arrest |
| J98 | 0.254 | 0.042 | 6.13 | Other respiratory disorders |
| I24 | 0.034 | 0.006 | 5.91 | Other acute ischemic heart diseases |
| T17 | 0.011 | 0.002 | 5.74 | Foreign body in respiratory tract |
| E77 | 0.001 | 0.000 | 5.46 | Disorders of glycoprotein metabolism |
| T47 | 0.001 | 0.000 | 5.46 | Agents primarily affecting the gastrointestinal system |
| G00 | 0.001 | 0.000 | 5.46 | Bacterial meningitis, not elsewhere classified |
| N17 | 0.531 | 0.098 | 5.40 | Acute kidney failure |
| T37 | 0.002 | 0.000 | 5.24 | Other systemic anti-infectives and antiparasitics |
| J81 | 0.026 | 0.005 | 5.14 | Pulmonary edema |
| E43 | 0.093 | 0.018 | 5.08 | Unspecified severe protein-calorie malnutrition |
| B33 | 0.003 | 0.001 | 4.92 | Other viral diseases, not elsewhere classified |
| J69 | 0.046 | 0.009 | 4.90 | Pneumonitis due to solids and liquids |
| G93 | 0.284 | 0.058 | 4.86 | Other disorders of brain |
| J94 | 0.009 | 0.002 | 4.82 | Other pleural conditions |
| T21 | 0.002 | 0.000 | 4.81 | Burn and corrosion of trunk |
| B49 | 0.007 | 0.002 | 4.81 | Unspecified mycosis |
| Y84 | 0.010 | 0.002 | 4.77 | Oth medical procedures cause abn react/compl, w/o misadvnt |
| I26 | 0.260 | 0.055 | 4.75 | Pulmonary embolism |
| B97 | 0.030 | 0.006 | 4.75 | Viral agents as the cause of diseases classified elsewhere |
| T36 | 0.010 | 0.002 | 4.71 | Systemic antibiotics |
| B44 | 0.005 | 0.001 | 4.68 | Aspergillosis |
| R43 | 0.030 | 0.007 | 4.52 | Disturbances of smell and taste |
| E44 | 0.068 | 0.015 | 4.48 | Protein-calorie malnutrition of moderate and mild degree |
| R78 | 0.056 | 0.013 | 4.40 | Find of drugs and oth substnc, not normally found in blood |
| J11 | 0.004 | 0.001 | 4.37 | Influenza due to unidentified influenza virus |
| J04 | 0.004 | 0.001 | 4.37 | Acute laryngitis and tracheitis |
| Y65 | 0.000 | 0.000 | 4.37 | Other misadventures during surgical and medical care |
| Y79 | 0.001 | 0.000 | 4.37 | Orthopedic devices associated with adverse incidents |
| A87 | 0.000 | 0.000 | 4.37 | Viral meningitis |
| J67 | 0.001 | 0.000 | 4.37 | Hypersensitivity pneumonitis due to organic dust |
| V18 | 0.000 | 0.000 | 4.37 | Pedal cycle rider injured in noncollision transport accident |
| D01 | 0.001 | 0.000 | 4.37 | Carcinoma in situ of other and unspecified digestive organs |
| V48 | 0.000 | 0.000 | 4.37 | Car occupant injured in noncollision transport accident |
| A81 | 0.000 | 0.000 | 4.37 | Atypical virus infections of central nervous system |
| B48 | 0.001 | 0.000 | 4.37 | Other mycoses, not elsewhere classified |
| R83 | 0.000 | 0.000 | 4.37 | Abnormal findings in cerebrospinal fluid |
| E86 | 0.147 | 0.034 | 4.36 | Volume depletion |
| J86 | 0.008 | 0.002 | 4.28 | Pyothorax |
| R50 | 0.200 | 0.047 | 4.26 | Fever of other and unknown origin |
| K72 | 0.034 | 0.008 | 4.24 | Hepatic failure, not elsewhere classified |
| B34 | 0.076 | 0.018 | 4.23 | Viral infection of unspecified site |
| T80 | 0.009 | 0.002 | 4.21 | Comp following infusion, transfusion and theraputc injection |
| I95 | 0.149 | 0.036 | 4.08 | Hypotension |
| B25 | 0.006 | 0.002 | 4.04 | Cytomegaloviral disease |
| R05 | 0.672 | 0.168 | 4.01 | Cough |
| R64 | 0.014 | 0.004 | 3.94 | Cachexia |
| F73 | 0.001 | 0.000 | 3.82 | Profound intellectual disabilities |
| E09 | 0.004 | 0.001 | 3.70 | Drug or chemical induced diabetes mellitus |
| B95 | 0.060 | 0.016 | 3.67 | Strep as the cause of diseases classified elsewhere |
| A40 | 0.004 | 0.001 | 3.64 | Streptococcal sepsis |
| A86 | 0.001 | 0.000 | 3.64 | Unspecified viral encephalitis |
| L89 | 0.110 | 0.030 | 3.62 | Pressure ulcer |
| D52 | 0.001 | 0.000 | 3.50 | Folate deficiency anemia |
| D62 | 0.107 | 0.031 | 3.47 | Acute posthemorrhagic anemia |

The 100 highest incidence ratios, comparing COVID positive persons to age, month and propensity-score matched comparators, are listed here. There was a total of 566 incidence ratios where the ratio was >1, indicating higher incidence in the COVID group than in the comparator group.
