# Supplementary material for: Using a data-driven approach to define post-COVID conditions in US electronic health record data
Source: PLoS One. 2024 Apr 5;19(4):e0300570. doi: 10.1371/journal.pone.0300570 (PMC10997091; doi:10.1371/journal.pone.0300570)
Supplement: S4 Table — (DOCX) [file pone.0300570.s004.docx]

# S4 Table: One Hundred Lowest Incidence Ratios, Comparing Persons with COVID to Age, Month and Propensity-Score Matched Persons

| ICD-10-CM Subchapter Code | Incidence in COVID positive group (%) | Incidence in comparator group (%) | Incidence Ratio | Description of ICD-10-CM Subchapter Code |
| --- | --- | --- | --- | --- |
| D03 | 0.000 | 0.003 | 0.10 | Melanoma in situ |
| Q68 | 0.000 | 0.002 | 0.11 | Other congenital musculoskeletal deformities |
| F93 | 0.001 | 0.004 | 0.12 | Emotional disorders with onset specific to childhood |
| M93 | 0.000 | 0.003 | 0.12 | Other osteochondropathies |
| E31 | 0.000 | 0.001 | 0.12 | Polyglandular dysfunction |
| S03 | 0.000 | 0.001 | 0.16 | Dislocation and sprain of joints and ligaments of head |
| D11 | 0.000 | 0.001 | 0.16 | Benign neoplasm of major salivary glands |
| M88 | 0.000 | 0.001 | 0.16 | Osteitis deformans [Paget's disease of bone] |
| S96 | 0.000 | 0.002 | 0.18 | Injury of muscle and tendon at ankle and foot level |
| W55 | 0.000 | 0.001 | 0.18 | Contact with other mammals |
| T15 | 0.000 | 0.001 | 0.18 | Foreign body on external eye |
| Q17 | 0.000 | 0.001 | 0.18 | Other congenital malformations of ear |
| Q20 | 0.001 | 0.003 | 0.19 | Congenital malformations of cardiac chambers and connections |
| L57 | 0.011 | 0.059 | 0.19 | Skin changes due to chronic expsr to nonionizing radiation |
| D66 | 0.000 | 0.002 | 0.20 | Hereditary factor VIII deficiency |
| F65 | 0.000 | 0.001 | 0.20 | Paraphilias |
| L94 | 0.000 | 0.002 | 0.20 | Other localized connective tissue disorders |
| R36 | 0.001 | 0.003 | 0.20 | Urethral discharge |
| Q52 | 0.000 | 0.001 | 0.20 | Other congenital malformations of female genitalia |
| L82 | 0.007 | 0.034 | 0.20 | Seborrheic keratosis |
| D23 | 0.002 | 0.009 | 0.21 | Other benign neoplasms of skin |
| D48 | 0.006 | 0.030 | 0.21 | Neoplasm of uncertain behavior of other and unsp sites |
| H34 | 0.002 | 0.008 | 0.21 | Retinal vascular occlusions |
| F64 | 0.003 | 0.016 | 0.21 | Gender identity disorders |
| H31 | 0.000 | 0.002 | 0.22 | Other disorders of choroid |
| W45 | 0.000 | 0.001 | 0.22 | Foreign body or object entering through skin |
| D04 | 0.002 | 0.007 | 0.22 | Carcinoma in situ of skin |
| C13 | 0.000 | 0.001 | 0.22 | Malignant neoplasm of hypopharynx |
| Q37 | 0.000 | 0.002 | 0.22 | Cleft palate with cleft lip |
| H52 | 0.007 | 0.030 | 0.22 | Disorders of refraction and accommodation |
| L59 | 0.001 | 0.002 | 0.23 | Oth disorders of skin, subcu related to radiation |
| M66 | 0.000 | 0.001 | 0.23 | Spontaneous rupture of synovium and tendon |
| T63 | 0.001 | 0.003 | 0.23 | Toxic effect of contact with venomous animals and plants |
| N23 | 0.000 | 0.001 | 0.23 | Unspecified renal colic |
| Q35 | 0.000 | 0.001 | 0.23 | Cleft palate |
| L56 | 0.000 | 0.001 | 0.23 | Other acute skin changes due to ultraviolet radiation |
| M92 | 0.001 | 0.004 | 0.23 | Other juvenile osteochondrosis |
| L66 | 0.001 | 0.002 | 0.23 | Cicatricial alopecia [scarring hair loss] |
| H21 | 0.001 | 0.002 | 0.24 | Other disorders of iris and ciliary body |
| K38 | 0.000 | 0.001 | 0.24 | Other diseases of appendix |
| Q62 | 0.001 | 0.002 | 0.24 | Congen defects of renal pelvis and congen malform of ureter |
| Q14 | 0.000 | 0.001 | 0.24 | Congenital malformations of posterior segment of eye |
| L80 | 0.001 | 0.003 | 0.24 | Vitiligo |
| K01 | 0.001 | 0.002 | 0.24 | Embedded and impacted teeth |
| R86 | 0.000 | 0.001 | 0.24 | Abnormal findings in specimens from male genital organs |
| L81 | 0.005 | 0.022 | 0.24 | Other disorders of pigmentation |
| D22 | 0.010 | 0.041 | 0.25 | Melanocytic nevi |
| Q76 | 0.001 | 0.003 | 0.25 | Congenital malformations of spine and bony thorax |
| Q04 | 0.001 | 0.005 | 0.25 | Other congenital malformations of brain |
| S33 | 0.001 | 0.004 | 0.26 | Disloc & sprain of joints & ligaments of lumbar spin & pelv |
| H15 | 0.001 | 0.003 | 0.26 | Disorders of sclera |
| B85 | 0.000 | 0.001 | 0.26 | Pediculosis and phthiriasis |
| M76 | 0.012 | 0.046 | 0.26 | Enthesopathies, lower limb, excluding foot |
| E30 | 0.001 | 0.005 | 0.26 | Disorders of puberty, not elsewhere classified |
| A54 | 0.001 | 0.003 | 0.26 | Gonococcal infection |
| N90 | 0.002 | 0.009 | 0.27 | Other noninflammatory disorders of vulva and perineum |
| H95 | 0.000 | 0.001 | 0.27 | Intraop and postproc comp and disorders of ear/mastd, NEC |
| L88 | 0.000 | 0.001 | 0.27 | Pyoderma gangrenosum |
| Q26 | 0.000 | 0.001 | 0.27 | Congenital malformations of great veins |
| Q13 | 0.000 | 0.001 | 0.27 | Congenital malformations of anterior segment of eye |
| Q67 | 0.001 | 0.005 | 0.27 | Congenital ms deformities of head, face, spine and chest |
| D41 | 0.000 | 0.001 | 0.27 | Neoplasm of uncertain behavior of urinary organs |
| G10 | 0.000 | 0.001 | 0.27 | Huntington's disease |
| H17 | 0.001 | 0.002 | 0.27 | Corneal scars and opacities |
| V86 | 0.000 | 0.001 | 0.27 | Occ off-road veh injured transp acc |
| C08 | 0.000 | 0.001 | 0.27 | Malignant neoplasm of other and unsp major salivary glands |
| A63 | 0.002 | 0.007 | 0.28 | Oth predominantly sexually transmitted diseases, NEC |
| L83 | 0.002 | 0.005 | 0.29 | Acanthosis nigricans |
| F80 | 0.014 | 0.049 | 0.29 | Specific developmental disorders of speech and language |
| D16 | 0.001 | 0.002 | 0.29 | Benign neoplasm of bone and articular cartilage |
| I78 | 0.001 | 0.003 | 0.30 | Diseases of capillaries |
| F54 | 0.001 | 0.003 | 0.30 | Psych & behavrl factors assoc w disord or dis classd elswhr |
| L84 | 0.005 | 0.018 | 0.30 | Corns and callosities |
| S43 | 0.008 | 0.026 | 0.30 | Disloc and sprain of joints and ligaments of shoulder girdle |
| C44 | 0.015 | 0.049 | 0.30 | Other and unspecified malignant neoplasm of skin |
| N81 | 0.008 | 0.027 | 0.31 | Female genital prolapse |
| D18 | 0.006 | 0.018 | 0.31 | Hemangioma and lymphangioma, any site |
| Q66 | 0.003 | 0.010 | 0.31 | Congenital deformities of feet |
| L24 | 0.001 | 0.002 | 0.31 | Irritant contact dermatitis |
| N75 | 0.001 | 0.003 | 0.31 | Diseases of Bartholin's gland |
| S56 | 0.001 | 0.002 | 0.31 | Injury of muscle, fascia and tendon at forearm level |
| E50 | 0.000 | 0.001 | 0.31 | Vitamin A deficiency |
| C52 | 0.000 | 0.001 | 0.31 | Malignant neoplasm of vagina |
| D42 | 0.000 | 0.001 | 0.31 | Neoplasm of uncertain behavior of meninges |
| Y99 | 0.002 | 0.006 | 0.32 | External cause status |
| B07 | 0.012 | 0.039 | 0.32 | Viral warts |
| F91 | 0.008 | 0.027 | 0.32 | Conduct disorders |
| N87 | 0.003 | 0.008 | 0.32 | Dysplasia of cervix uteri |
| H00 | 0.002 | 0.007 | 0.32 | Hordeolum and chalazion |
| F07 | 0.003 | 0.010 | 0.32 | Personality & behavrl disorders due to known physiol cond |
| S16 | 0.004 | 0.012 | 0.32 | Injury of muscle, fascia and tendon at neck level |
| D31 | 0.001 | 0.002 | 0.33 | Benign neoplasm of eye and adnexa |
| L20 | 0.009 | 0.028 | 0.33 | Atopic dermatitis |
| N41 | 0.003 | 0.008 | 0.33 | Inflammatory diseases of prostate |
| A64 | 0.001 | 0.003 | 0.33 | Unspecified sexually transmitted disease |
| L64 | 0.001 | 0.004 | 0.33 | Androgenic alopecia |
| V43 | 0.000 | 0.001 | 0.34 | Car occupant injured pick-up truck, pick-up truck or van |
| Q39 | 0.000 | 0.001 | 0.34 | Congenital malformations of esophagus |
| L60 | 0.014 | 0.043 | 0.34 | Nail disorders |
| N60 | 0.008 | 0.025 | 0.34 | Benign mammary dysplasia |

The 100 lowest incidence ratios, comparing COVID positive persons to age, month and propensity-score matched comparators, are listed here. There was a total of 775 incidence ratios where the ratio was <1, indicating lower incidence in the COVID group than in the comparator group.
