# Supplementary material for: Using a data-driven approach to define post-COVID conditions in US electronic health record data
Source: PLoS One. 2024 Apr 5;19(4):e0300570. doi: 10.1371/journal.pone.0300570 (PMC10997091; doi:10.1371/journal.pone.0300570)
Supplement: S5 Table — (DOCX) [file pone.0300570.s005.docx]

# S5 Table: Comparison of ICD-10-CM code U09.9 to Data-Driven Definition

| Persons diagnosed with COVID in September 2021 | Data-driven PCC  present in follow-up | Data-driven PCC  absent in follow-up |  |
| --- | --- | --- | --- |
| U09.9 code  present in follow-up | 394 | 538 | 2.9% of cases diagnosed in September |
| U09.9 code  absent in follow-up | 5,716 | 25,538 |  |
|  | 19.0% of cases diagnosed in September |  |  |
