# Supplementary material for: Using a data-driven approach to define post-COVID conditions in US electronic health record data
Source: PLoS One. 2024 Apr 5;19(4):e0300570. doi: 10.1371/journal.pone.0300570 (PMC10997091; doi:10.1371/journal.pone.0300570)
Supplement: S6 Table — (DOCX) [file pone.0300570.s006.docx]

# S6 Table: Comparison of Label Consistency Via K-Nearest Neighbor Analysis

|  | Nearest Neighbor Consistency |
| --- | --- |
| Data-driven Post-COVID Conditions present | 99.7% |
| Data-driven Post-COVID Conditions absent | 98.6% |
| U09.9 code present | 98.9% |
| U09.9 code absent | 62.7% |

The class label of a given person was compared with the class label of their nearest neighbor in the multivariate patient characteristics space.
