# Supplementary material for: Using a data-driven approach to define post-COVID conditions in US electronic health record data
Source: PLoS One. 2024 Apr 5;19(4):e0300570. doi: 10.1371/journal.pone.0300570 (PMC10997091; doi:10.1371/journal.pone.0300570)
Supplement: S7 Table — (DOCX) [file pone.0300570.s007.docx]

# S7 Table: Results of Sensitivity Analyses

|  | **Population size** | **Persons with Post-COVID Conditions** | **Incidence of PCC** |
| --- | --- | --- | --- |
| Main analysis: Using ICD-10-CM codes at subchapter level. | 588,611 | 118,018 | 20.1% |
| Using ICD-10-CM codes at level of precision in record rather than subchapters. | 588,611 | 120,167 | 20.4% |
| No longer excluding codes that indicate “history of” and ICD-10 Z codes. | 588,611 | 142,000 | 24.1% |

Incidence of PCC was compared when computing symptomatology for different resolution of ICD-10-CM encoding; subchapter-level (e.g. J12.89 was encoded as J12), diagnostic record entry (e.g. J12.89 itself), as well as retaining codes for “history of” and Z codes.
