# Supplementary material for: Using a data-driven approach to define post-COVID conditions in US electronic health record data
Source: PLoS One. 2024 Apr 5;19(4):e0300570. doi: 10.1371/journal.pone.0300570 (PMC10997091; doi:10.1371/journal.pone.0300570)
Supplement: S1 Fig — (DOCX) [file pone.0300570.s008.docx]

# S1 Figure: COVID Case Attack Rate

**
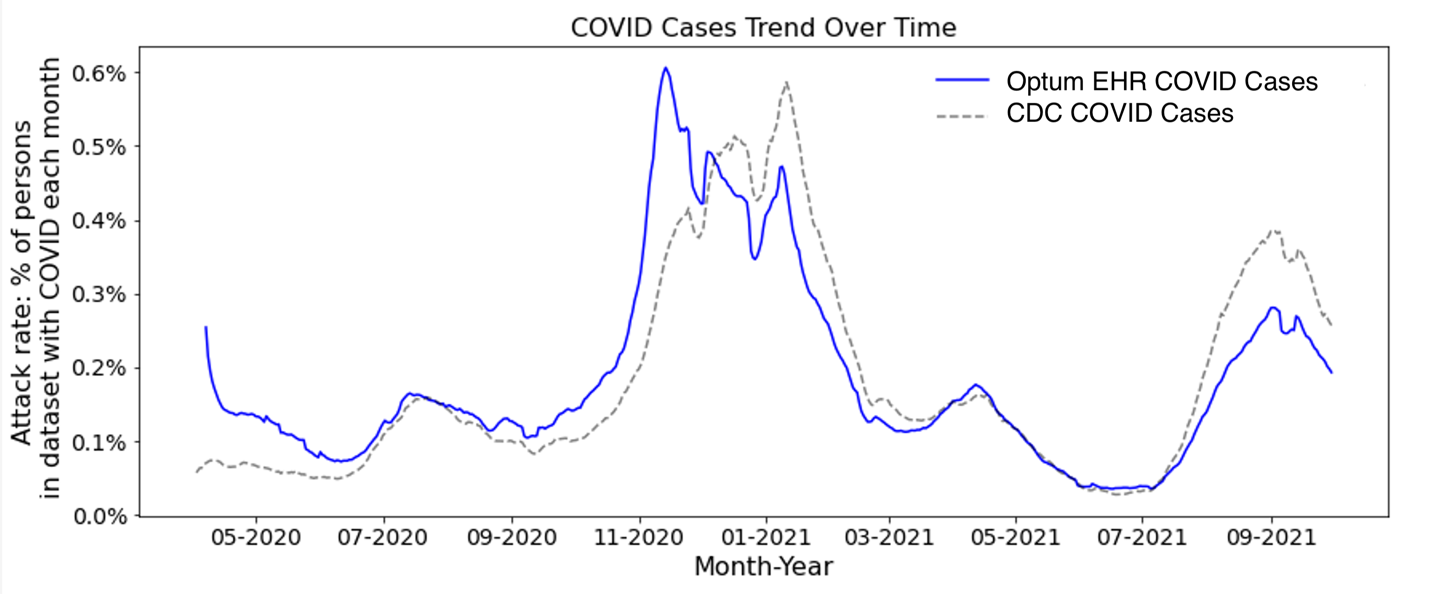
**

Comparison of COVID case attach rate within Optum® EHR data and CDC reports. 7-day rolling average was plotted for both datasets. Peak cross-correlation between the two trend lines was 0.86 (where 1 is perfect) with a shift of 40 days.
