# Supplementary material for: Using a data-driven approach to define post-COVID conditions in US electronic health record data
Source: PLoS One. 2024 Apr 5;19(4):e0300570. doi: 10.1371/journal.pone.0300570 (PMC10997091; doi:10.1371/journal.pone.0300570)
Supplement: S2 Fig — (DOCX) [file pone.0300570.s009.docx]

# S2 Figure: Total Variational Distance Measures of Post-COVID Conditions and U09.9 Populations


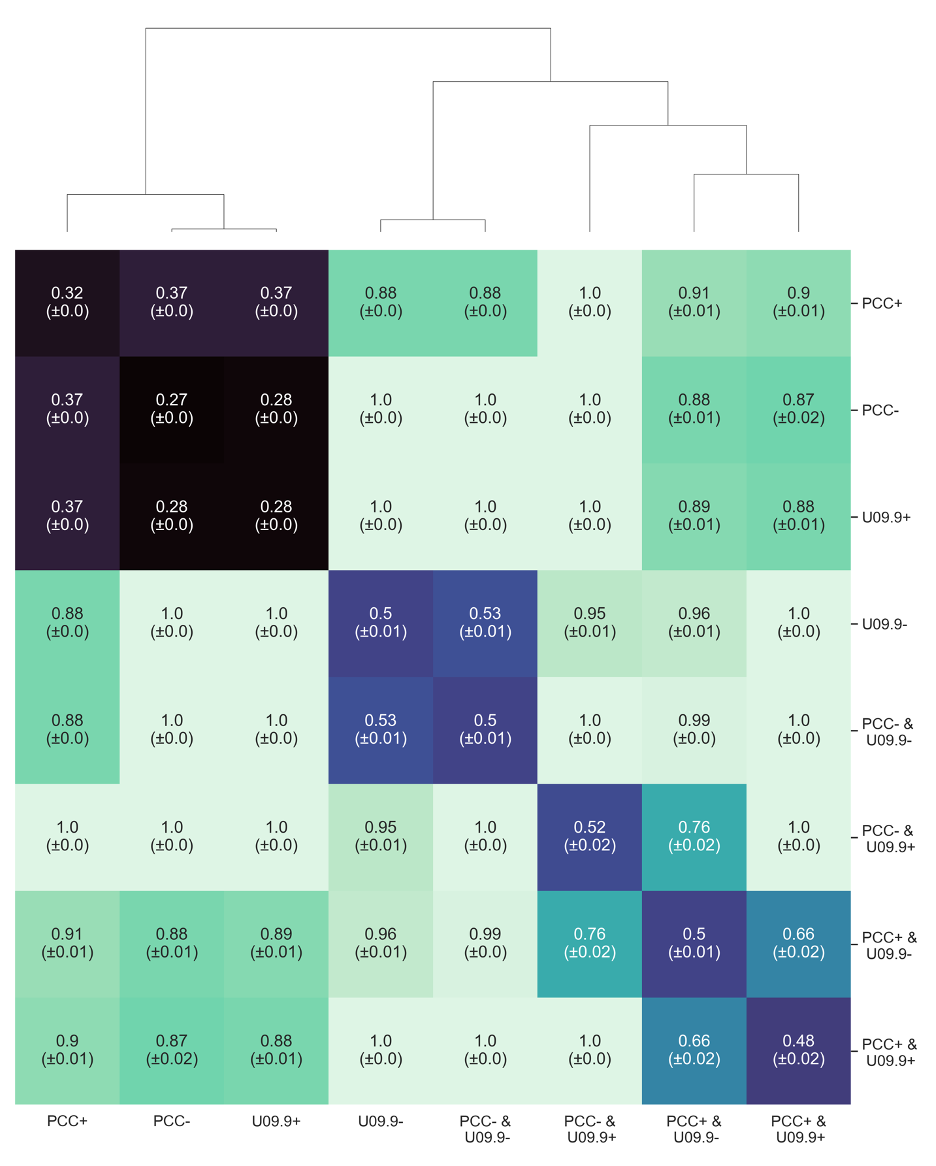


PCC+: Post-COVID Conditions present, by data-driven definition

PCC-: Post-COVID Conditions absent, by data-driven definition

U09.9+: ICD-10-CM U09.9 code present

U09.9-: ICD-10-CM U09.9 code absent
