# Supplementary figures and images for: Using a data-driven approach to define post-COVID conditions in US electronic health record data
Source: PLoS One. 2024 Apr 5;19(4):e0300570. doi: 10.1371/journal.pone.0300570 (PMC10997091; doi:10.1371/journal.pone.0300570)

# S3 Figure: Symptomatology Comparison Between Persons with Post-COVID Conditions and U09.9 Codes


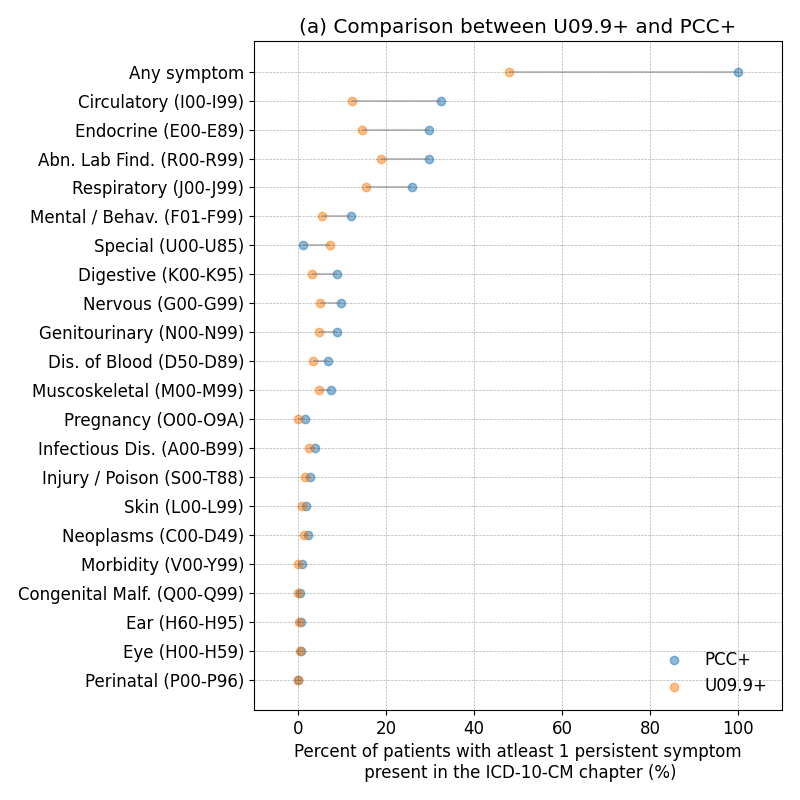

Supplement: S3 Fig — (DOCX) [file pone.0300570.s010.docx]
