# Supplementary material for: Using a data-driven approach to define post-COVID conditions in US electronic health record data
Source: PLoS One. 2024 Apr 5;19(4):e0300570. doi: 10.1371/journal.pone.0300570 (PMC10997091; doi:10.1371/journal.pone.0300570)
Supplement: S4 Fig — (DOCX) [file pone.0300570.s011.docx]

# S4 Figure: Time to Next Encounter in Optum® EHR Before and After COVID Diagnosis

**
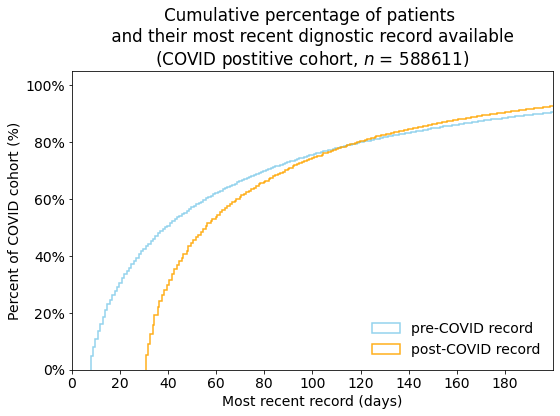
**

Plot indicates that 50% of patients have post-COVID diagnostic records (orange) within 60 days of Sars-COV-2 infection, and 30 days after the acute period, while 50% of patients have pre-COVID diagnostic records up to 30 days prior to Sars-COV-2 infection.
